# Exploration of the relationship between tumor-infiltrating lymphocyte score and histological grade in breast cancer

**DOI:** 10.1186/s12885-024-12069-0

**Published:** 2024-03-07

**Authors:** Deyong Kang, Chuan Wang, Zhonghua Han, Liqin Zheng, Wenhui Guo, Fangmeng Fu, Lida Qiu, Xiahui Han, Jiajia He, Lianhuang Li, Jianxin Chen

**Affiliations:** 1 Department of Pathology, Fujian Medical University Union Hospital, 350001 Fuzhou, P. R. China; 2 Breast Surgery Ward, Department of General Surgery, Fujian Medical University Union Hospital, 350001 Fuzhou, P. R. China; 3 Key Laboratory of OptoElectronic Science and Technology for Medicine of Ministry of Education, Fujian Provincial Key Laboratory of Photonics Technology, College of Photonic and Electronic Engineering, Fujian Normal University, 350007 Fuzhou, P. R. China; 4 College of Physics and Electronic Information Engineering, Minjiang University, 350108 Fuzhou, P. R. China; 5 School of Science, Jimei University, 361021 Xiamen, P. R. China

**Keywords:** Tumor-infiltrating lymphocyte score, Multiphoton microscopy, Histological grade

## Abstract

**Background:**

The histological grade is an important factor in the prognosis of invasive breast cancer and is vital to accurately identify the histological grade and reclassify of Grade2 status in breast cancer patients.

**Methods:**

In this study, data were collected from 556 invasive breast cancer patients, and then randomly divided into training cohort (*n* = 335) and validation cohort (*n* = 221). All patients were divided into actual low risk group (Grade1) and high risk group (Grade2/3) based on traditional histological grade, and tumor-infiltrating lymphocyte score (TILs-score) obtained from multiphoton images, and the TILs assessment method proposed by International Immuno-Oncology Biomarker Working Group (TILs-WG) were also used to differentiate between high risk group and low risk group of histological grade in patients with invasive breast cancer. Furthermore, TILs-score was used to reclassify Grade2 (G2) into G2 /Low risk and G2/High risk. The coefficients for each TILs in the training cohort were retrieved using ridge regression and TILs-score was created based on the coefficients of the three kinds of TILs.

**Results:**

Statistical analysis shows that TILs-score is significantly correlated with histological grade, and is an independent predictor of histological grade (odds ratio [OR], 2.548; 95%CI, 1.648–3.941; *P* < 0.0001), but TILs-WG is not an independent predictive factor for grade (*P* > 0.05 in the univariate analysis). Moreover, the risk of G2/High risk group is higher than that of G2/Low risk group, and the survival rate of patients with G2/Low risk is similar to that of Grade1, while the survival rate of patients with G2/High risk is even worse than that of patients with G3.

**Conclusion:**

Our results suggest that TILs-score can be used to predict the histological grade of breast cancer and potentially to guide the therapeutic management of breast cancer patients.

**Supplementary Information:**

The online version contains supplementary material available at 10.1186/s12885-024-12069-0.

## Introduction

The histological grade of breast cancer is a well-recognized clinical variable, and the Nottingham grading system, modified by Elston and Ellis of Broome, is the most widely adopted grading classification system [1, 2]. The histological grade obtained from each slide is based on the degree of tubule formation, nuclear pleomorphism and mitotic counts. A numerical assessment of each criterion was carried out to classify the histological grade into 3 levels, namely, low grade (Grade1), intermediate grade (Grade2), and high grade (Grade3) [3]. Grade1 tumors are well-differentiated, have a low risk of recurrence and can be treated more conservatively; Grade2 tumors are moderately differentiated; and Grade 3 tumors are poorly differentiated, associated with a poor prognosis and should be treated more aggressively [1, 4]. Clinically, about half of breast cancer is classified as Grade1 or Grade3 status, but a considerable proportion is classified as Grade2 (30 − 60%). The concordance of breast cancer grading by pathologists shows that high consistency can be observed in Grade1 (Kappa value: 0.51) and Grade3 (Kappa value: 0.60), while low consistency was observed with Grade 2 (Kappa value: 0.33). Therefore, Grade2 cannot be used as a basis for clinical decision-making due to the intermediate risk of recurrence and low consistency [5, 6]. Furthermore, Engstrøm et al. reclassified all cases of breast cancer into six subtypes by gene expression analysis, and found that differences in breast cancer specific survival according to the subtypes occurred almost exclusively amongst patients with Grade2 tumors [7]. Previous studies have shown that histological grade, tumor size, degree of axillary lymph node (LN) involvement, age, hormone receptor status, HER2/neu status and the presence of lymphovascular invasion (LVI) were the prognostic factors for breast cancer [8]. The histological grade that considers both morphology and proliferation has unique prognostic significance compared to other prognostic factors and was widely used in clinical decision-making [4]. In addition, the histological grade has been incorporated into a variety of validated prognostic algorithms to determine the treatment of breast cancer, such as the Nottingham Prognostic Index, Adjuvant! Online and St Gallen guidelines [9–11]. Therefore, accurate identification of the histological grade of invasive breast cancer and refinement of Grade2 status have high clinical implications.

In recent decades, an increasing number of studies have focused on the prognostic impact of tumor microenvironment (TME), especially tumor-infiltrating lymphocytes (TILs) has been widely investigated as a prognostic and predictive biomarker in breast cancer [12–14]. Miyoshi et al. showed that for the ER+/HER2- (ER+: estrogen receptor positive) breast cancer, high levels of TILs predicted a shorter survival time after recurrence, and that the proportion of TILs was significantly correlated with histological grade [15]. Another study showed that increased TILs was associated with an excellent prognosis in node-positive, ER-/HER2- (ER-: estrogen receptor negative) breast cancer and that TILs was associated with high histological grade [16]. Many similar studies showed that TILs were associated with the histological grade of tumors [17–19]. In our previous study, the percentage of TILs, which is based on the consensus recommendation proposed by International Immuno-Oncology Biomarker Working Group (TILs-WG), was obtained from the hematoxylin and eosin (H&E)-stained sections to assess its prognostic value [20], but have not been utilized to investigate its relationship with histological grade and TILs.

Since its development in 1990, multiphoton microscopy (MPM) combining second harmonic generation (SHG) and two-photon excitation fluorescence (TPEF) signals has become an important imaging modality in biomedical sciences [21]. It can be applied to the non-invasive study of biological samples to obtain three-dimensional imaging with sub-micron resolution [22]. TPEF signals can be detected from endogenous fluorophores such as nicotinamide adenine dinucleotide (NADH), flavin adenine dinucleotide (FAD), and porphyrin, while SHG signals can be detected from non-centrosymmetric molecules such as collagen. Thus, MPM is able to simultaneously image cellular and extracellular matrix structures for label-free analysis of tissue samples [23]. It is considered one of the best non-invasive means of performing bioimaging in tissues and live animals, with the following advantages: (1) since multiphoton absorption occurs only at the focal point of the objective, MPM provides optical sectioning capability and avoids out-of-focus light bleaching; (2) the use of near-infrared (NIR) wavelengths allows for low scattering and deep tissue imaging [24]. The TPEF and SHG signals provide enhanced contrast and facilitate image interpretation, therefore, MPM has been widely developed and applied in the biomedical science field with the development of interdisciplinary medicine [25, 26].

In our previous study, MPM was used to image TILs in the TME of breast cancer and obtained a tumor-infiltrating lymphocyte score (TILs-score) for each patient, and statistical analysis showed that TILs-score was an independent prognostic factor for breast cancer [27]. We also improved the prognostic value by combining the TILs-score and TILs-WG in breast cancer [20]. In this work, we further investigated the relationship between TILs-WG, TILs-score and histological grade. The discrimination ability of the TILs-score was analyzed by the area under the curve (AUC), and a nomogram model combining the TILs-score with the clinical factors was developed for personalized prediction of histological grade in breast cancer patients.

## Materials and methods

### Study population and sample preparation

**Fig. 1:**
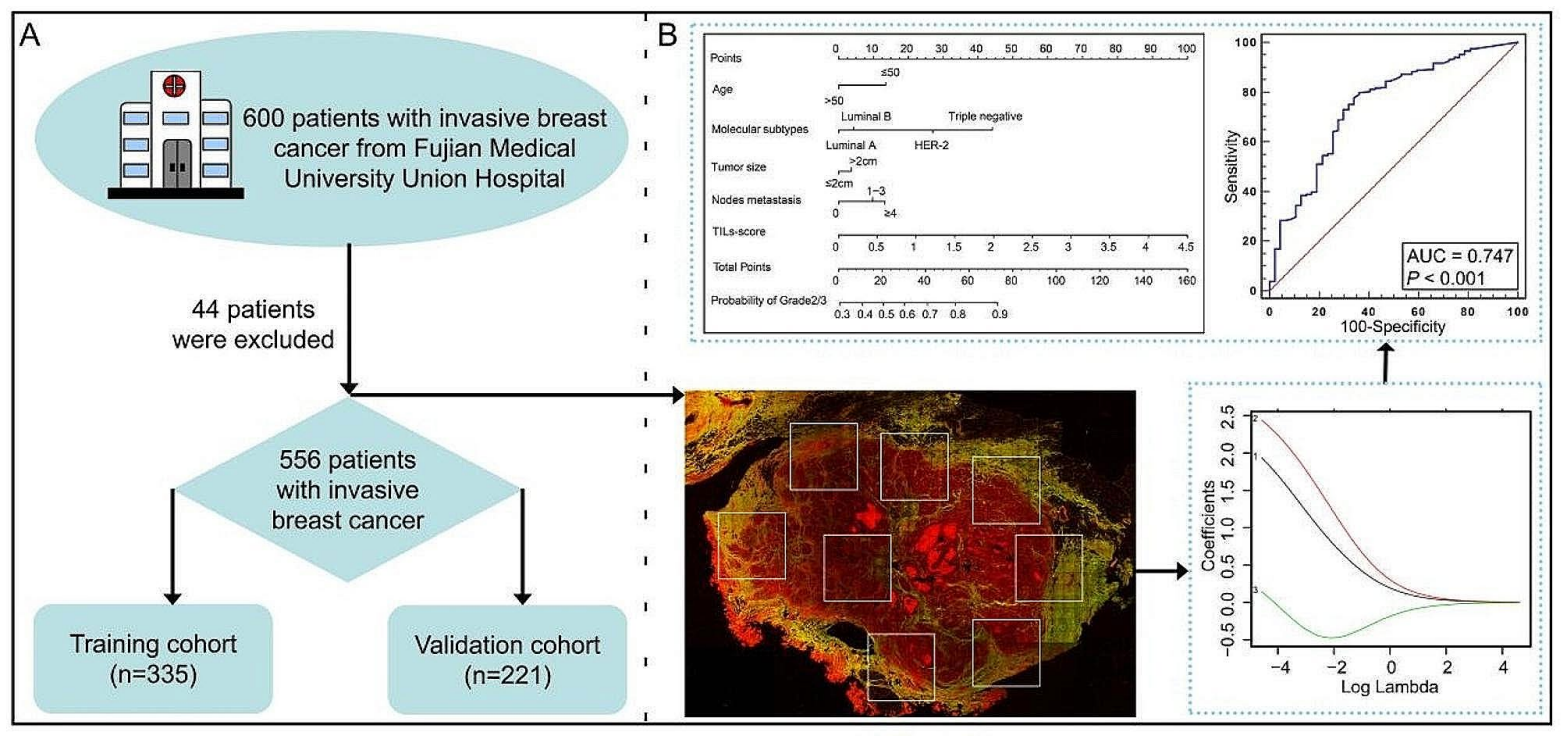
A flowchart to display the selection of patients **A** and experimental scheme **B**

This retrospective study was approved by the Institutional Review Boards of Fujian Medical University Union Hospital. A total of 600 patients who were diagnosed with invasive breast cancer participated in our study. 44 patients were excluded due to neoadjuvant chemotherapy and radiotherapy, sections without lymphocytes, tumors located in lymph nodes, no available histological grade information and pathological reports, and 556 patients who passed quality control were included in this study and randomly segregated into the training cohort (*n* = 335) and validation cohort (*n* = 221), as shown in Fig. 1A. Tumor histological grade was retrospectively assessed according to the Nottingham system and Elston-Ellis grading method [1, 2], and is also treated as an ordinal categorical variable (Grade1/low grade, 13.49%; Grade2/intermediate grade, 51.08%; Grade3/high grade, 35.43%). In this study, all patients were divided into actual low risk group (Grade1) and high risk group (Grade2/3) based on traditional histological grade. The clinicopathologic characteristics of patients are shown in Table 1. Two continuous slices (5 μm thickness) were cut from each formalin-fixed paraffin-embedded (FFPE) tissue block. One slice was stained by H&E, and the other was used for MPM imaging.

**Table 1:** Characteristics of patients with breast cancer in actual low and high risk groups

Characteristics	Training cohort (*n* = 335)		P-value	Validation cohort (*n* = 221)		P-value
	Low risk (Grade1) (*n* = 47)	High risk (Grade2/3) (*n* = 288)		Low risk (Grade1) (*n* = 28)	High risk (Grade2/3) (*n* = 193)	
**Age**			0.265			0.975
≤ 50	23 (48.9%)	166 (57.6%)		15 (53.6%)	104 (53.9%)	
> 50	24 (51.1%)	122 (42.4%)		13 (46.4%)	89 (46.1%)	
**Molecular subtype**			< 0.0001			< 0.0001
Luminal A	26 (48.9%)	58 (20.1%)		16 (57.1%)	37 (19.2%)	
Luminal B	16 (34.0%)	86 (29.9%)		5 (17.9%)	71 (36.8%)	
HER2-enriched	5 (10.6%)	73 (25.3%)		4 (14.3%)	42 (21.8%)	
Triple-negative	3 (6.4%)	71 (24.7%)		3 (10.7%)	43 (22.3%)	
**Tumor size**			0.015			0.03
≤ 2 cm	29 (61.7%)	123 (42.7%)		16 (57.1%)	69 (35.8%)	
> 2 cm	18 (32.3%)	165 (57.3%)		12 (42.9%)	124 (64.2%)	
**Nodes metastasis**			0.187			0.465
0	31 (66.0%)	149 (51.7%)		16 (57.1%)	94 (48.7%)	
1–3	9 (19.1%)	73 (25.3%)		7 (25.0%)	43 (22.3%)	
≥ 4	7 (14.9%)	66 (22.9%)		5 (17.9%)	56 (29.0%)	
**ER**			< 0.0001			0.096
Negative	8 (17%)	142 (49.3%)		7 (25%)	80 (41.5%)	
Positive	39 (83%)	146 (50.7%)		21 (75%)	113 (58.5%)	
**PR**			< 0.0001			0.036
Negative	12 (25.5%)	155 (53.8%)		9 (32.1%)	103 (53.4%)	
Positive	35 (74.5%)	133 (46.2%)		19 (67.9%)	90 (46.6%)	
**HER2**			0.242			0.321
Negative	34 (72.3%)	183 (63.5%)		22 (78.6%)	134 (69.4%)	
Positive	13 (27.7%)	105 (36.5%)		6 (21.4%)	59 (30.6%)	
**TILs-WG** **median (IQR)**	10% (5-10%)	10% (5-20%)	0.457	10% (6.25-15%)	10% (10-20%)	0.018
**TILs-score median (IQR)**	1.335 (0.738-2.10)	2.194 (1.767–2.704)	< 0.0001	1.428 (0.749–2.036)	2.219 (1.652–2.750)	< 0.0001

*Abbreviations* ER, estrogen receptor; PR, progesterone receptor; IQR, interquartile range

### Multiphoton imaging system and data acquisition

The imaging system has been described in detail in the previous publication [28]. In short, it is based on a commercially upright laser scanning microscope (LSM 880, Zeiss, Germany) combined with a mode-locked femtosecond Ti: sapphire laser (Chameleon Ultra, Coherent, USA), tunable from 690 to 1064 nm. The excitation wavelength was set to 810 nm for all experiments, the SHG signal was collected from 395 to 415 nm (green color) by a GaAsP PMT, and the TPEF signal was collected from 428 to 695 nm (red color) by a 32-channel GaAsP PMT array detector. A Plan-Apochromat ×20 objective (NA = 0.8, Zeiss, Germany) was used to obtain high-resolution imaging.

The method of quantifying TILs-WG and TILs-score have been described in detail in previous studies [20, 27]. Simply put, the percentage of TILs in breast cancer was assessed separately by two pathologists on H&E-stained sections according to the standard method proposed by the International Immuno-Oncology Biomarker Working Group, and then the average percentage was taken as the final TILs percentage (TILs-WG) for each patient [20]. MPM can identify cells (such as tumor cells and lymphocytes) and extracellular matrix structures (such as collagen fibers) by endogenous signals. In the study, we first obtained the MPM images of each sample based on the 7–25 non-overlapping regions of interest (ROIs) marked on the H&E images within the tumor nest, tumor boundary, and invasive front, and one ROI may have multiple types of TILs, and a kind of TILs could exist in multiple ROIs. Then we observed the relative spatial positions of tumor cells, TILs, and collagen fibers, and classified the TILs in the TME into three patterns, namely TILs1-3, and recorded their occurrence frequencies separately. As shown in Supplementary Fig. 1, TILs-1 is defined as a pattern of infiltrating lymphocytes surrounded by tumor cells, TILs-2 is defined as a pattern of infiltrating lymphocytes around tumor cells, and TILs-3 is defined as infiltrating lymphocytes distributed in the TME without direct contact with tumor cells. Finally, we retrieved the coefficients for each TILs using ridge regression with cross validation based on the occurrence frequency of TILs1-3 in the training cohort, and fixed the coefficients of three kinds of TILs in a formula to calculate a patient-specific TILs score, which was used to study the relationship between TILs and the grade of breast cancer as shown in Fig. 1B.

### Statistical analysis

The relationship between traditional clinical risk factors, TILs-score and histological grade (Grade1 group and Grade2/3 group) was explored using univariate and multivariate logistic regression analyses. A clinical (CLI) model based on the four clinicopathological factors (age, molecular subtype, tumor size, nodes metastasis) was developed and used to predict histological grade. The discrimination was measured by the AUC of the receiver operating characteristics (ROC) curve. A nomogram was created in the software package R using the nomogram function from the rms library. The nomogram created in the training cohort was applied to the validation cohort and the regression coefficients of the variables in the multiple regression were scaled to a score of 0-100. The variable with the largest regression coefficient had the greatest impact and was assigned a score of 100. The scores for each variable were summed to give a total score which was transformed into predicted probabilities. The calibration curve was drawn to evaluate the predictive ability of the nomogram model. The optimal cutoff value calculated from the training cohort was used to classify patients into low risk group and high risk group, and determine the sensitivity (SEN), specificity (SPE), positive predictive value (PPV), and negative predictive value (NPV) of the models, which was also applied to the validation cohort. The TILs-score was analyzed with Mann-Whitney U test, and differences between categorical variables were compared using the χ^2^ test. In order to estimate the association between histological grade and patients’ survival time, a Kaplan-Meier survival analysis was conducted and compared by log-rank test. The above statistical analyses were carried out using R 3.6.3, IBM SPSS Statistics 25 and Graph-Pad Prism 6.0.

## Results

### Clinicopathologic characteristics of patients

We included 556 patients with a median age of 49 years old (range, 24–84 years old). The clinicopathologic characteristics of training and validation cohorts are shown in Supplementary Table 1. There are no significant differences between the two cohorts, as well as the distribution of TILs-WG and TILs-score (*P* > 0.05). In addition, the molecular subtype, tumor size, progesterone receptor (PR) and TILs-score between the low risk and high risk groups are significantly different in both the training and validation cohorts (*P* < 0.05), as shown in Table 1.

### Prediction of histological grade using TILs-score

We first analyzed the relationship between histological grade and patient DFS and the results were shown in Supplementary Fig. 2. We found that there is a difference in survival rates between Grade1 and Grade2 patients, the survival rates of patients with Grade1 are better than Grade2 (hazard ratio [HR], 1.935; 95% confidence interval [CI], 0.9467 to 3.167; *P* = 0.0759). Similarly, survival rates are different between Grade1 and Grade3 patients, patients with Grade1 have better survival rates than Grade3 (HR, 1.97; 95% CI, 0.9438 to 3.383; *P* = 0.0755) in the training cohort, although the differences are not statistically significant, but there is almost no difference in survival between Grade2 and Grade3 patients (HR, 1.013; 95% CI, 0.6633 to 1.548; *P* = 0.9415). While in the validation cohort, there is almost no difference between Grade1 and Grade2 (HR, 0.8386; *P* = 0.6222) and Grade3 (HR, 1.064; *P* = 0.865), and a slight difference in survival rates between Grade2 and Grade3 patients (HR, 1.28; *P* = 0.3276). Furthermore, a Kaplan-Meier survival analysis was performed on the whole cohort, and the results are consistent with that of the training cohort, with the differences between Grade1 and Grade2 (HR, 1.329; 95% CI, 0.8167 to 2.073; *P* = 0.2695) and Grade3 (HR, 1.478; 95% CI, 0.8911 to 2.299; *P* = 0.1391), and no difference between Grade2 and Grade3 (HR, 1.116; 95% CI, 0.8079 to 1.548; *P* = 0.5018), despite all of these differences have not statistically significance. The HR values, 95% CI, and *P* values are summarized in Supplementary Table 2. Therefore, all patients are divided into actual low risk group (Grade1) and high risk group (Grade2/3) based on traditional histological grade.

**Table 2:** Univariate and multivariate logistic regression analyses of the association of variables with pathologic grades in the training cohort

Characteristics	Univariate analysis				Multivariate analysis			
	OR	(95%CI)		P-value	OR	(95%CI)		P-value
**Age**								
≤ 50	Reference							
> 50	0.704	0.38	1.306	0.266	NA			NA
**Molecular subtype**								
Luminal A	Reference							
Luminal B	2.131	1.038	4.378	0.039	1.320	0.603	2.892	0.487
HER2-enriched	5.79	2.074	16.164	0.001	3.291	1.121	9.660	0. 030
Triple-negative	9.385	2.683	32.827	< 0.0001	5.644	1.552	20.526	0.009
**Tumor size**								
≤ 2 cm	Reference							
> 2 cm	2.161	1.148	4.069	0.017	NA			NA
**Nodes metastasis**								
0	Reference							
1–3	1.688	0.763	3.73	0.196	NA			NA
≥ 4	1.962	0.822	4.682	0.129	NA			NA
**ER**								
Negative	Reference							
Positive	0.21	0.095	0.467	< 0.0001	NA			NA
**PR**								
Negative	Reference							
Positive	0.294	0.147	0.590	0.001	NA			NA
**HER2**								
Negative	Reference							
Positive	1.501	0.758	2.970	0.244	NA			NA
**TILs-WG**	1.027	0.997	1.058	0.078	NA			NA
**TILs-score**	2.987	2.019	4.417	< 0.0001	2.577	1.696	3.917	< 0.0001

*Abbreviations* OR, odds ratio; ER, estrogen receptor; PR, progesterone receptor

**Fig. 2:**
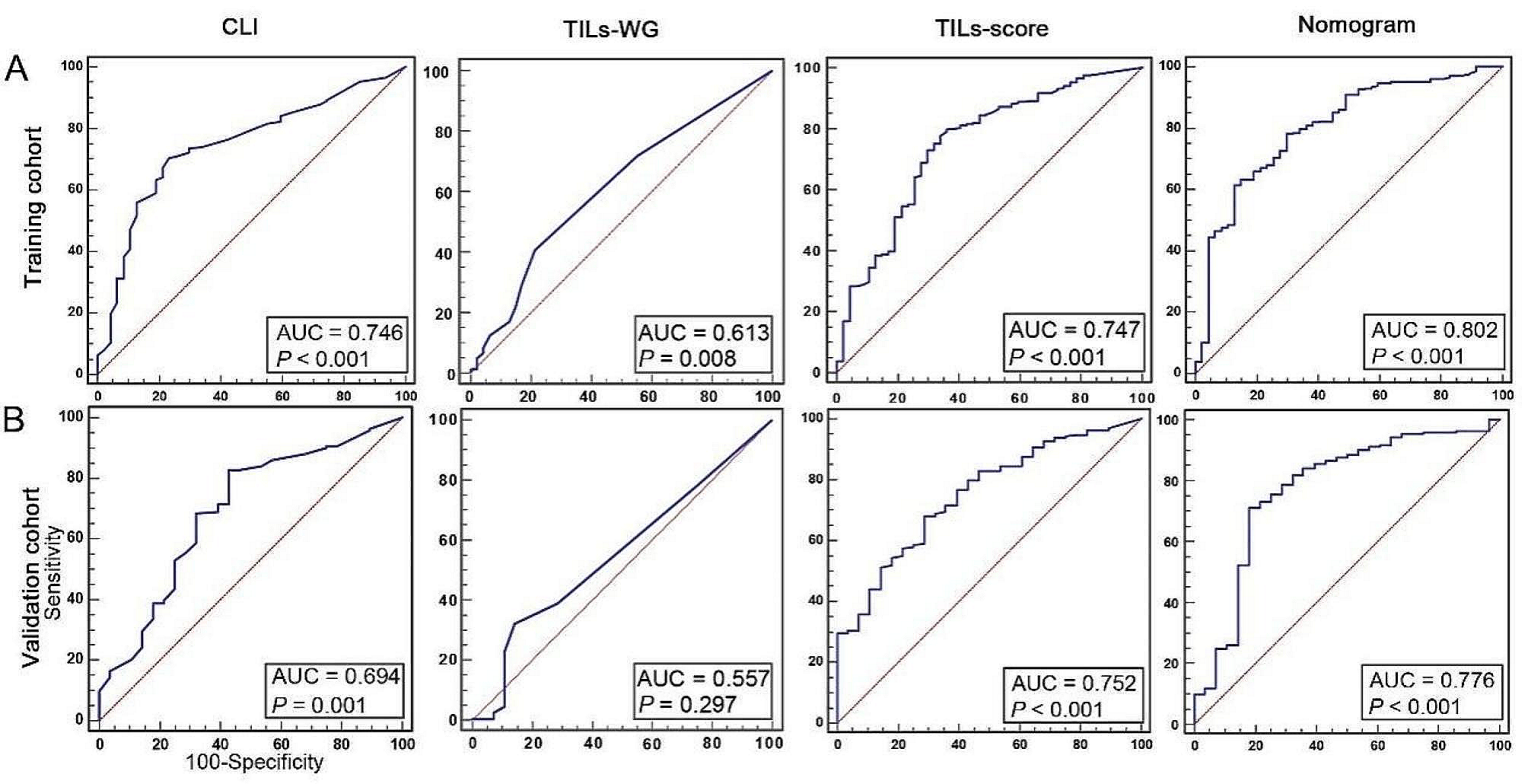
The ROC curves of the CLI, TILs-WG, TILs-score and nomogram models in the training **A** and validation cohorts **B**

The correlations of histological grade with clinical factors (age, molecular subtype, tumor size, nodes metastasis), expression of receptors or proteins on the surface of breast cancer cells (ER; PR; HER2), TILs-WG and TILs-score in the training cohort were assessed by the logistic regression analysis. Table 2 shows the results of the univariate and multivariate logistic regression analyses. The univariate logistic regression analysis reveals a significant association of histological grade with molecular subtype, tumor size, ER, PR and TILs-score (*P* < 0.05), while the correlation between TILs-WG and histologic grade is not statistically significant (odds ratio [OR], 1.027; 95% CI, 0.997 to 1.058; *P* = 0.078). After incorporating the factors that are significantly correlated with grade in the univariate logistic regression analysis into the multivariate analysis, the results show that TILs-score proves to be a strong independent predictor of histological grade (OR, 2.577; 95% CI, 1.696 to 3.917; *P* < 0.0001). To further evaluate the predictive accuracy of TILs-score, we estimated the AUC of TILs-score by the ROC analysis, and a CLI model which combines age, molecular subtype, tumor size, nodes metastasis was also developed to compare. As shown in Fig. 2A, the CLI model shows an AUC of 0.746 (95% CI, 0.696 to 0.792) and TILs-score shows an AUC of 0.747 (95% CI, 0.697 to 0.793) for predicting the low and high risk groups in the training cohort. The results indicate that the predictive ability of TILs-score is equivalent to that of the CLI model with four factors. What’s more, the AUC of TILs-score (AUC, 0.752; 95% CI, 0.689 to 0.807) is higher than that of CLI model (AUC, 0.694; 95% CI, 0.628 to 0.754) in the validation cohort (Fig. 2B). While the predictive ability of TILs-WG is not better than that of TILs-score and CLI model in both the training and validation cohorts (Training cohort: AUC = 0.613, 95% CI, 0.559 to 0.665; validation cohort: AUC = 0.557, 95% CI, 0.489 to 0.623). Since TILs-WG is not an independent predictive factor for histological grade (*P* > 0.05), we will not analyze the relationship between TILs-WG and grade in the following analysis.

**Fig. 3:**
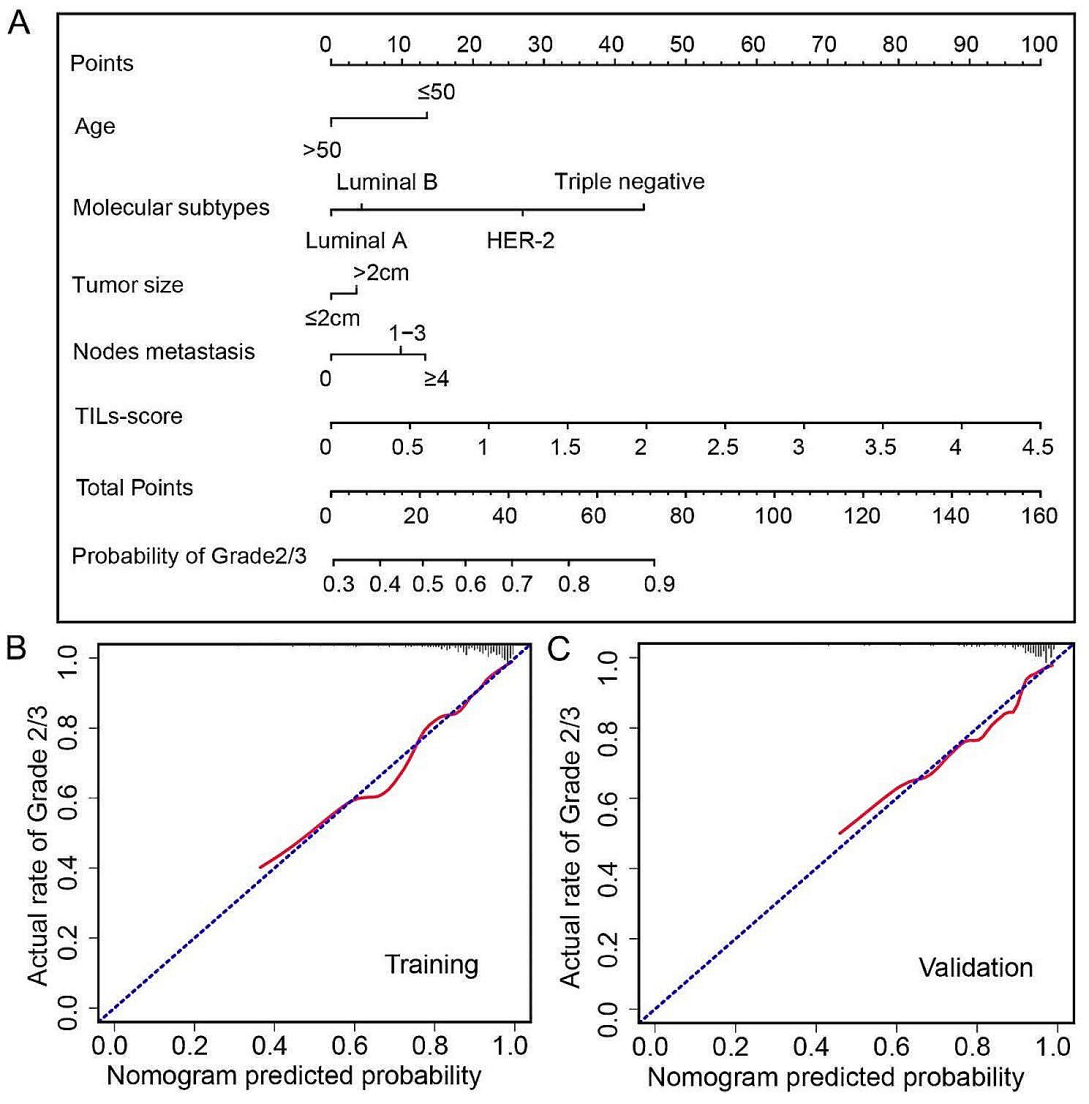
**A** The nomogram combining the TILs-score with clinical factors in the training cohort. **B** The calibration curves of the nomogram in the training cohort. **C** The calibration curves of the nomogram in the validation cohort

Furthermore, a nomogram which combines TILs-score with clinical factors is built for individualized prediction of low and high risk groups, and can increase the AUC of CLI model from 0.746 (95% CI, 0.696 to 0.792) to 0.802 (95% CI, 0.755 to 0.843) in the training cohort, and from 0.694 (95% CI, 0.628 to 0.754) to 0.776 (95% CI, 0.715 to 0.829) in the validation cohort, respectively. As displayed in Fig. 3, TILs-score has the greatest contribution to the prediction of histological grade. Several subgroup analyses are also conducted to assess the predictive performance of TILs-score. As shown in Table 3, TILs-score performs well except for HER2-enriched, triple-negative, and ER-negative patients. This is likely because TILs-score is more appropriate for assessing patients with ER-positive breast cancer rather than ER-negative breast cancer, as demonstrated by our previous works [20, 27].

**Table 3:** Prediction of clinicopathologically classified patients by the TILs-score

Subgroups	Predicted low risk (*N* = 155)	Predicted high risk (*N* = 401)	OR (95%)	P-value	AUC (95%)	SEN (95%)	SPE (95%)
**Age**							
≤ 50	92 (59.4%)	216 (53.9%)	3.182 (2.059–4.916)	< 0.0001	0.763 (0.712–0.810)	75.56 (70.0-80.6)	68.42 (51.3–82.5)
> 50	63 (30.6%)	185 (46.1%)	2.833 (1.806–4.442)	< 0.0001	0.739 (0.679–0.792)	80.09 (74.1–85.3)	56.76 (39.5–72.9)
**Molecular subtype**							
Luminal A	66 (42.6%)	68 (17.0%)	2.736 (1.652–4.531)	< 0.0001	0.730 (0.647–0.803)	62.11 (51.6–71.9)	76.92 (60.7–88.9)
Luminal B	36 (23.2%)	142 (35.4%)	3.139 (1.720–5.729)	< 0.0001	0.745 (0.675–0.808)	83.44 (76.7–88.9)	52.38 (29.8–74.3)
HER2-enriched	30 (19.4%)	94 (23.4%)	1.316 (0.565–3.065)	0.525	0.558 (0.467–0.648)	76.52 (67.7–83.9)	33.33 (7.5–70.1)
Triple-negative	23 (14.8%)	97 (24.2%)	2.283 (0.859–6.066)	0.098	0.633 (0.540–0.719)	82.46 (74.2–88.9)	50.0 (11.8–88.2)
**Tumor size**							
≤ 2 cm	86 (55.5%)	151 (37.7%)	2.372 (1.609–3.497)	< 0.0001	0.716 (0.654–0.773)	71.35 (64.4–77.6)	68.89 (53.4–81.8)
> 2 cm	69 (44.5%)	250 (62.3%)	3.729 (2.175–6.391)	< 0.0001	0.760 (0.710–0.806)	81.31 (76.3–85.6)	53.33 (34.3–71.7)
**Nodes metastasis**							
0	89 (57.4%)	201 (50.1%)	2.922 (1.970–4.334)	< 0.0001	0.750 (0.696–0.798)	76.13 (70.3–81.3)	65.96 (50.7–79.1)
1–3	31 (20.0%)	101 (25.2%)	3.383 (1.685–6.792)	0.001	0.778 (0.698–0.846)	81.90 (73.7–88.4)	62.50 (35.4–84.8)
≥ 4	35 (22.6%)	99 (24.7%)	2.533 (1.190–5.392)	0.016	0.701 (0.616–0.777)	75.41 (66.8–82.8)	50.00 (21.1–78.9)
**ER**							
Negative	50 (32.3%)	187 (46.6%)	1.693 (0.889–3.224)	0.109	0.598 (0.533–0.661)	79.73 (73.8–84.8)	40.00 (16.3–67.7)
Positive	105 (67.7%)	214 (53.4%)	3.229 (2.223–4.692)	< 0.0001	0.770 (0.720–0.815)	74.90 (69.2–80.1)	68.33 (55.0–79.7)
**PR**							
Negative	63 (40.6%)	216 (53.9%)	1.939 (1.117–3.366)	0.019	0.636 (0.576–0.692)	79.07 (73.6–83.9)	42.86 (21.8–66.0)
Positive	92 (59.4%)	185 (46.1%)	3.260 (2.195–4.843)	< 0.0001	0.775 (0.721–0.823)	75.78 (69.6–81.3)	70.37 (56.4–82.0)
**HER2**							
Negative	112 (72.3%)	261 (65.1%)	3.400 (2.338–4.945)	< 0.0001	0.778 (0.732–0.819)	76.97 (71.9–81.5)	69.64 (55.9–81.2)
Positive	43 (27.7%)	140 (34.9%)	2.010 (1.127–3.585)	0.018	0.660 (0.587–0.729)	78.66 (71.6–84.7)	42.11 (20.3–66.5)

*Abbreviations*: OR, odds ratio; AUC, area under the curve; SEN, sensitivity; SPE, specificity

### Performance comparison of different prediction models

**Fig. 4:**
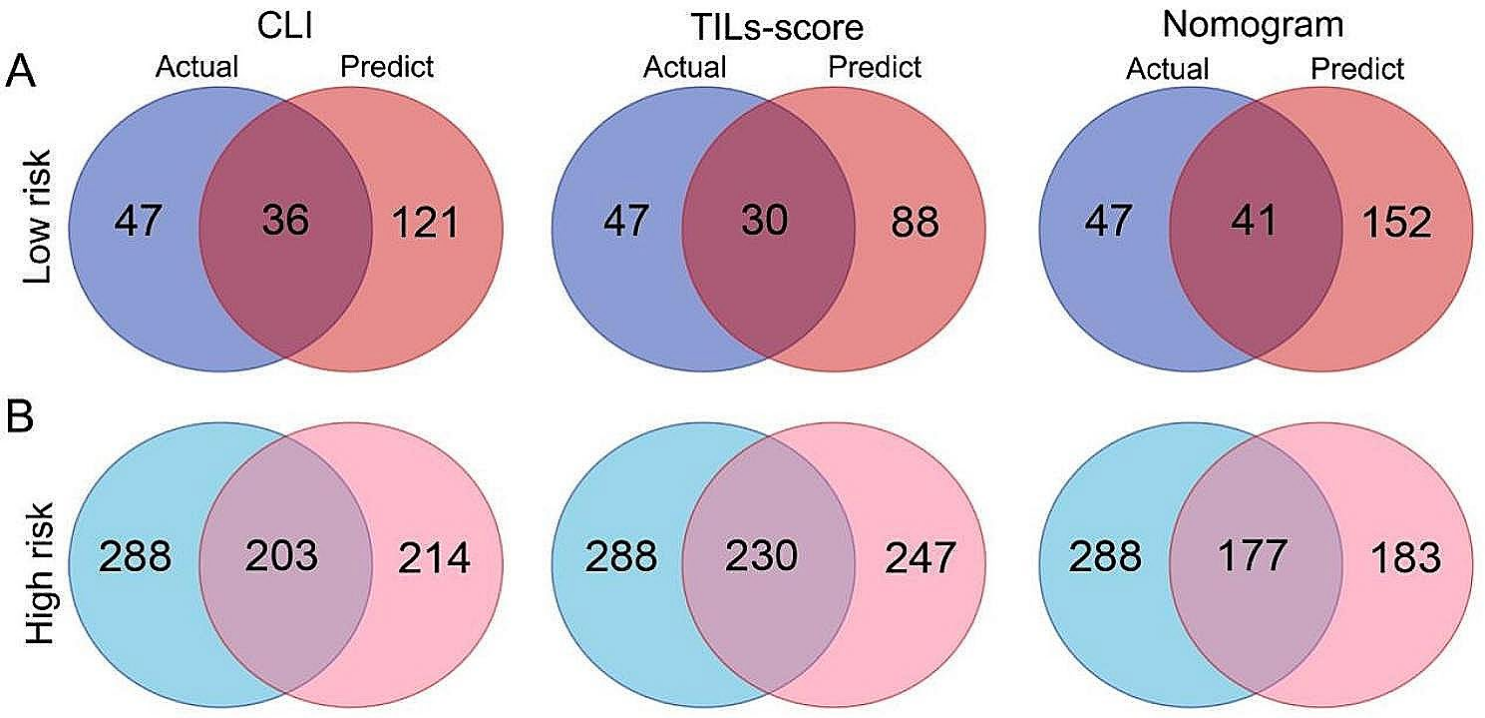
**A** Venn software was used to identify the overlapping patients between the actual and predicted in the low risk group of training cohort. **B** Venn software was used to identify the overlapping patients between the actual and predicted in the high risk group of training cohort

All patients are divided into the predicted low risk group and high risk group according to the optimal cutoff values obtained by the Youden index criterion from the training cohort. As shown in Table 1, the distribution of TILs-score between the actual low risk and high risk groups is significantly different in both the training and validation cohorts (*P* < 0.0001), and the median of TILs-score in the high risk is higher than that low risk group in both the training cohort (2.194 vs. 1.335) and validation cohort (2.219 vs. 1.428). In contrast, there is no significant difference in the distribution of TILs-WG in the actual low risk and high risk groups. Venn software is used to identify the overlapping patients between the actual and predicted, as shown in Fig. 4 and Supplementary Fig. 3. For example, in the training cohort, the actual number of patients diagnosed with low risk (Grade 1) is 47 and the predicted low risk of the CLI model is 121, of which 36 patients are actual low risk patients (Fig. 4A, left panel). While in the high risk (Grade 2/3) group, the overlapping number of patients between the actual and predicted is 203 (Fig. 4B, left panel). In both the training and validation cohorts, the number of overlapping patients of the TILs-score is comparable to that of the CLI model in both low risk and high risk groups (Fig. 4 and Supplementary Fig. 3, middle panel). The results show that TILs-score has a better predictive efficacy in the high risk group.

As shown in Table 4, the SEN, SPE, PPV and NPV of the CLI model are 70.49% (95% CI, 64.9 to 75.7), 76.60% (95% CI, 62.0 to 87.7), 94.86% (95% CI, 91.6 to 96.9) and 29.75% (95% CI, 25.0 to 35.0) in the training cohort, respectively. The SEN, SPE, PPV and NPV of the TILs-score are 79.17% (95% CI, 74.0 to 83.7), 63.83% (95% CI, 48.5 to 77.3), 93.06% (95% CI, 90.1 to 95.2) and 33.33% (95%CI, 26.8 to 40.6) in the training cohort, respectively. It can be found that the predictive performance of the TILs-score is comparable to that of the CLI model. The AUC, SPE and PPV of the nomogram model are higher than those of the CLI model and TILs-score (Table 4). The same analyses are performed in the validation cohort and similar results are observed (Supplementary Table 3).

**Table 4:** Performance comparison of different models for predicting pathological grades in the training cohort

Model	AUC (95%)	SEN (95%)	SPE (95%)	PPV (95%)	NPV (95%)
CLI	0.746 (0.696–0.792)	70.49 (64.9–75.7)	76.60 (62.0-87.7)	94.86 (91.6–96.9)	29.75 (25.0–35.0)
TILs-WG	0.613 (0.559–0.665)	40.63 (34.9–46.5)	78.72 (64.3–89.3)	92.1 (86.9–95.4)	17.8 (15.3–20.5)
TILs-score	0.747 (0.697–0.793)	79.17 (74.0-83.7)	63.83 (48.5–77.3)	93.06 (90.1–95.2)	33.33 (26.8–40.6)
Nomogram	0.802 (0.755–0.843)	61.46 (55.6–67.1)	87.23 (74.3–95.2)	96.72 (93.3–98.4)	26.97 (23.5–30.7)

*Abbreviations* AUC, area under the curve; SEN, sensitivity; SPE, specificity; PPV, positive predictive value; NPV, negative predictive value

### Reclassification of Grade2 using TILs-score

**Fig. 5:**
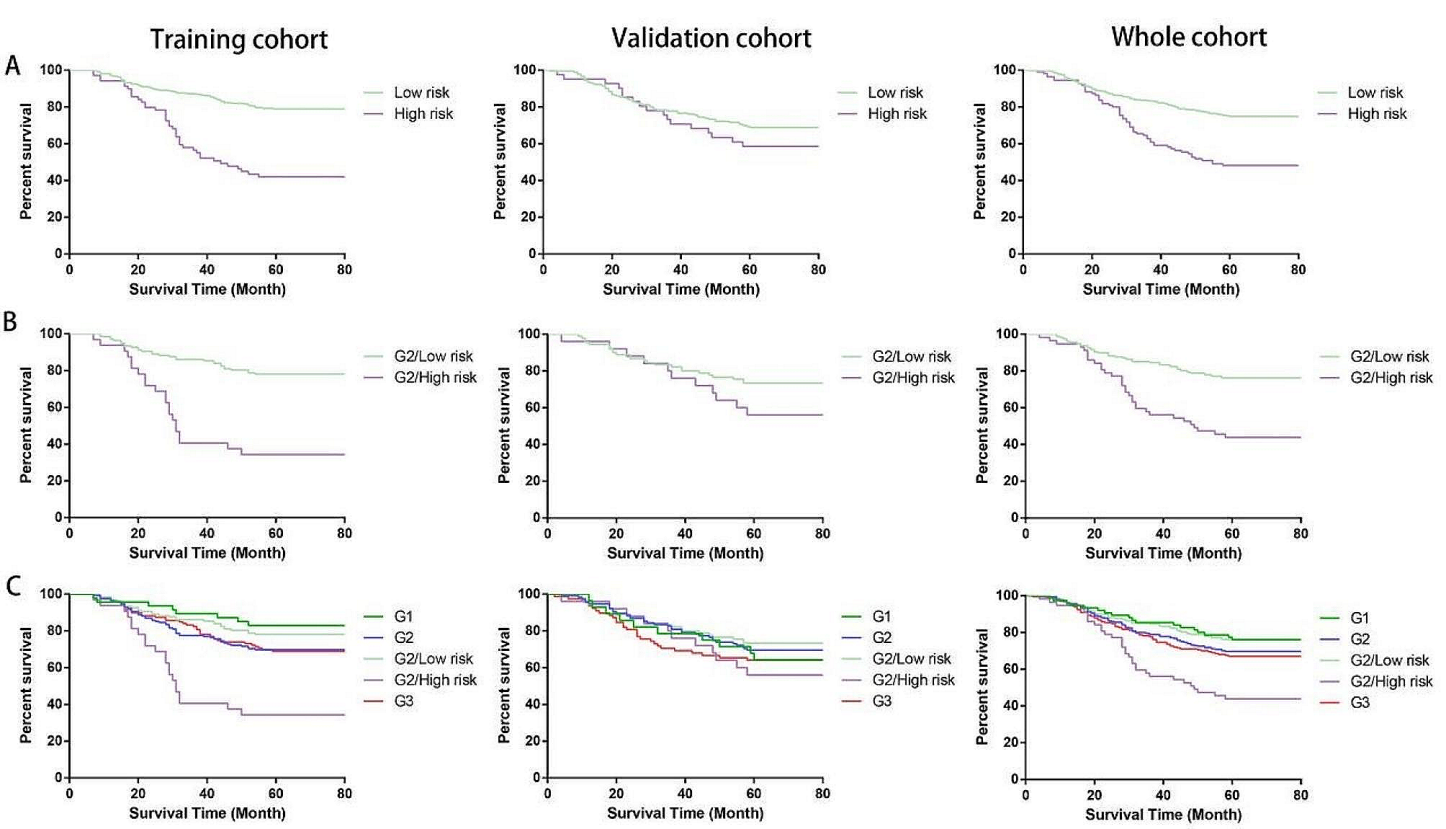
**A** Kaplan-Meier curves for the patients re-stratified into low and high risk group by TILs-score in the three cohorts. **B** Kaplan-Meier curves for the re-stratified G2 cases by TILs-score. **C** Kaplan-Meier curves comparing the G2/High-risk and G2/Low-risk groups with clinical histological grade in the three cohorts

We find that there are no differences in survival between Grade2 and Grade3 in the training cohort and in the whole cohort, and no difference in survival between Grade1 and Grade2 in the validation cohort. It appears that some patients with Grade2 have the same survival rate as patients with Grade1, while others have the same survival rate as patients with Grade3, as shown in Supplementary Fig. 2. Therefore, we tried to reclassify the patients into low risk and high risk groups using TILs-score, and as shown in Fig. 5A, it is found that patients in the high risk group have lower survival rate than patients in the low risk group. We also used TILs-score to reclassify patients with Grade2 into Grade2/High risk (G2/High risk) and Grade2/Low risk (G2/Low risk) and the survival rate of patients with Grade2 is significantly different (Fig. 5B). In the training cohort, patients in G2/High risk group have 4.09 fold higher risks for an event than patients in G2/Low risk group (HR, 4.09; 95% CI, 4.019 to 19.1; *P* < 0.0001), and the difference is statistically significant. Similarly, in the validation cohort and the whole cohort, patients in G2/High risk group have worse outcomes than patients in G2/Low risk group. In addition, we further used Kaplan-Meier survival curves to examine the correlation between G2/High risk group (G2/Low risk group) and clinical histological grade (Grade1, Grade2, Grade3). As shown in Fig. 5C, there is no significant difference in survival between G2/Low risk group and Grade1 in the training cohort (HR, 1.326; 95% CI, 0.6317 to 2.686; *P* = 0.4751), in the validation cohort (HR, 0.7291; 95% CI, 0.3203 to 1.571; *P* = 0.3983) and in the whole cohort (HR, 1.004; 95% CI, 0.5890 to 1.710; *P* = 0.9895). The survival rate of patients with G2/High risk is similar to Grade3 in validation cohort, and no significant difference in survival is found (HR, 1.153; 95% CI, 0.5642 to 2.386; *P* = 0.6871), but the survival rate of patients with G2/High risk is lower than that of patients with Grade3 in both the training cohort and the whole cohort (Training cohort: HR, 2.933; 95% CI, 2.237 to 9.042; *P* < 0.0001; Whole cohort: HR, 1.964; 95% CI, 1.378 to 3.723; *P* = 0.0014). The HR and *P* values of the three cohorts are shown in Table 5.

**Table 5:** Hazard ratios for 5-year DFS between different groups in the three cohorts

Comparison	Training cohort		Validation cohort		Whole cohort	
	HR	95%CI P-value	HR	95%CI P-value	HR	95%CI P-value
High risk Vs. Low risk	3.49	3.458–10.04 (< 0.0001)	1.372	0.7825–2.575 (= 0.2505)	2.411	2.109–4.670 (< 0.0001)
G2/High risk Vs. G2/Low risk	4.09	4.019–19.01 (< 0.0001)	1.733	0.8401–4.285 (0.1247)	2.881	2.409–7.410 (< 0.0001)
G2/Low risk Vs. G1	1.326	0.6317–2.686 (= 0.4751)	0.7291	0.3203–1.571 (= 0.3983)	1.004	0.5890–1.710 (= 0.9895)
G2/High risk Vs. G3	2.933	2.237–9.042 (< 0.0001)	1.153	0.5642–2.386 (= 0.6871)	1.964	1.378–3.723 (= 0.0014)

*Abbreviations* HR, hazard ratio; G1, Grade1; G2, Grade2; G3, Grade3

## Discussion

Breast cancer is a complex and heterogeneous disease as reflected by different morphological presentations, molecular features and responses to treatment. The routine clinical management of breast cancer relies on the availability of clinicopathological prognostic and predictive factors to support clinicians in their decision-making. Studies have shown that the main determinants of survival in breast cancer are tumor size, lymph node status and histological grade. Thereinto, histological grade represents a morphological assessment of the biological characteristics of tumor and has been shown to provide important information related to the clinical behaviors of breast cancer [29, 30]. The higher histological grade is associated with the lower overall survival and long-term survival [31, 32]. Galimberti et al. stated that histological grade was a significant predictor of disease-free survival [33], and Rakha et al. showed that histological grade was an independent prognostic factor in invasive lobular carcinoma of breast cancer [34]. In addition to the accurate assessment of histopathological features on H&E staining by the morphology of cells, imaging techniques such as X-ray mammography [35], magnetic resonance imaging (MRI) [36], and ultrasonography [37] can be used to predict histopathological features of breast cancer, but the resolution of these imaging methods is still limited. Currently, classification prediction for histological grade using artificial intelligence is also available, but the performance is affected by poorer consistency of tumors in the intermediate grade (Grade2) [38]. At present, the histological grade of tumors mainly refers to a semi-quantitative evaluation of the morphological characteristics of the tumor cells.

Many studies have shown that the microenvironment surrounding the tumor seed plays a crucial role in tumor growth and development [12]. TILs as an important component of TME has been shown to be an independent prognostic factor in breast cancer [16, 17]. Our previous study revealed that TILs-score obtained by MPM was an independent prognostic factor of breast cancer [27], and in this study, we further confirm the relationship between the TILs-score and the histological grade, and higher TILs-score is associated with a higher grade. Multivariate logistic regression analysis shows that TILs-score is an independent predictor of histological grade (OR, 2.577; *P* < 0.0001) and has comparable predictive power to the CLI model combining four factors (training cohort, AUC, 0.747 vs. 0.746; validation cohort, AUC, 0.752 vs. 0.694). Desmedt et al. demonstrated that only histological grade and proliferation modules correlated with relapse-free survival (RFS) in ER-positive/HER2-negative breast cancer [39]. In LN-negative [40, 41] or LN-positive [42, 43] breast cancer irrespective of ER expression and patients with ER-positive breast cancer without [44] or with neoadjuvant endocrine therapy [45], histological grade was an independent prognostic factor. Additionally, histological grade could complement the LN stage as it can influence the outcome of patients in different LN stage categories [43]. TILs-core was further used to divide patients into the low risk and high risk groups based on the optimal cutoff values, and over 90% of patients were predicted to be high risk who were truly high risk (training cohort, PPV, 93.06%, 95%CI, 90.1 to 95.2; validation cohort, PPV, 92.80%, 95%CI, 89.0 to 95.4). More importantly, the nomogram model which combined the TILs-score with clinical factors would increase the PPV of the CLI model from 94.86% (95% CI, 91.6 to 96.9) to 96.72% (95% CI, 93.3 to 98.4) in the training cohort and from 93.6% (95% CI, 89.5 to 96.2) to 96.0% (95% CI, 91.6 to 98.2) in the validation cohort, respectively. The results show a better predictive performance in the high risk group. The reason for the low NPV of all models may be because the number of patients actually diagnosed as low risk in the dataset is too small to provide a relevant prediction.

A study has shown that high-grade tumors carry a risk of early recurrence and death, and require consideration of timely use of adjuvant chemotherapy, while patients with low-grade tumors are almost invariably ER-positive and can be offered long-term follow-up with or without potentially less toxic systemic therapies. (i.e. endocrine therapy) [29]. However, up to half of breast cancer cases are categorized as “Grade2” in routine clinical practice, which may include a number of low grade (Grade1) and high grade (Grade2) tumors, so Grade2 is judged to be an intermediate risk group with limited clinical value [46]. As a result, many researchers opened several investigations at the patients with Grade2, for example, Sotiriou et al. used genes to investigate histological grade and found that gene expression grade indices correlate with Grades1 and Grade3, whereas the index for Grade2 spans the values of Grade1 and Grade3, and patients with Grade2 can be reclassified into low and high risk group via the index to improve the prognostic value [5]; similarly, Ivshina et al. used gene expression signatures for classification prediction of grade and the results showed that they could accurately classify Grade1 and Grade3, and could classify Grade2 tumors into two highly distinguishable categories (Grade2a and Grade2b genetic grades) whose survival outcomes were highly similar to those of Grade1 and Grade3, respectively [47]; Li et al. developed a qualitative transcriptional signature using within-sample relative expression orderings of gene pairs to redefine Grade1 and Grade3, and Grade2 is further reclassified into the redefined Grade1 and Grade3 [48]; Wang et al. reclassified the Nottingham histological grade 2 (NHG2) into DeepGrade2-low (DG2-low) and DG2-high groups, and found that DG2-high had an increased risk of recurrence compared to DG2-low, and besides DG2-low also had a similar phenotype to NHG1 and DG2-high with NHG3 [4]. In this work, TILs-score was used to reclassify G2 into G2 /Low risk and G2/High risk, and the results showed that TILs-score can classify G2 tumors into two highly distinguishable categories, and the risk of G2/High risk group is higher than that of G2/Low risk group. Moreover, the survival rate of patients with G2/Low risk is similar to that of patients with pathologically-determined histological grade1 (G1), while the survival rate of patients with G2/High risk is worse than that of patients with G3. Our findings are similar to those of previous studies, suggesting that patients with G2 should be re-stratified to screen out the high risk from low risk patients, prevent some patients from undertreatment or overtreatment, reduce the side effects and costs of chemotherapy, and improve patient survival and the quality of life.

## Conclusions

In summary, our results reveal that TILs-score obtained from MPM images is a simple method to differentiate the histological grade of breast cancer (Grade1 and Grade2/3). In addition, TILs-score could be used to re-stratify intermediate risk breast cancer patients (Grade2) to increase the information needed for clinical decision making, and thereby improve the survival rate and prognosis of breast cancer patients.

### Electronic supplementary material

Below is the link to the electronic supplementary material.

Supplementary Material 1

## Data Availability

Data are available from the corresponding author on reasonable request.

## Abbreviations

LN: lymph node
LVI: lymphovascular invasion
TME: tumor microenvironment
TILs: tumor-infiltrating lymphocytes
H&E: hematoxylin and eosin
MPM: multiphoton microscopy
SHG: second harmonic generation
TPEF: two-photon excitation fluorescence
NADH: nicotinamide adenine dinucleotide
FAD: flavin adenine dinucleotide
NIR: near-infrared
TILs-score: tumor-infiltrating lymphocyte score
AUC: area under the curve
FFPE: formalin-fixed paraffin-embedded
ROI: region of interest
CLI: clinical
ROC: receiver operating characteristics
SEN: sensitivity
SPE: specificity
PPV: positive predictive value
NPV: negative predictive value
DFS: disease-free survival
HR: hazard ratio
CI: confidence interval
OR: odds ratio
MRI: magnetic resonance imaging
RFS: relapse-free survival
