# Supplementary material for: Exploration of the relationship between tumor-infiltrating lymphocyte score and histological grade in breast cancer
Source: BMC Cancer. 2024 Mar 7;24:318. doi: 10.1186/s12885-024-12069-0 (PMC10921807; doi:10.1186/s12885-024-12069-0)
Supplement: Supplementary file 1 — Supplementary Material 1 [file 12885_2024_12069_MOESM1_ESM.docx]

Supplementary materials for

Exploration of the relationship between tumor-infiltrating lymphocyte score and histological grade in breast cancer

Deyong Kang,^1,†^ Chuan Wang,^2,†^ Zhonghua Han,^2,†^ Liqin Zheng,^3^ Wenhui Guo,^2^ Fangmeng Fu,^2^ Lida Qiu,^4^ Xiahui Han,^3^ Jiajia He,^5,*^ Lianhuang Li,^3,*^ and Jianxin Chen^3,*^

^1^Department of Pathology, Fujian Medical University Union Hospital, Fuzhou 350001, P. R. China

^2^Breast Surgery Ward, Department of General Surgery, Fujian Medical University Union Hospital, Fuzhou 350001, P. R. China

^3^Key Laboratory of OptoElectronic Science and Technology for Medicine of Ministry of Education, Fujian Provincial Key Laboratory of Photonics Technology, College of Photonic and Electronic Engineering, Fujian Normal University, Fuzhou 350007, P. R. China

^4^College of Physics and Electronic Information Engineering, Minjiang University, Fuzhou 350108, P. R. China

^5^School of Science, Jimei University, Xiamen 361021, P. R. China

^†^These authors contributed equally to this work.

***Corresponding author:**

Jiajia He, School of Science, Jimei University, Xiamen 361021, P. R. China. E-mail: [hejiajia@jmu.edu.cn;](mailto:hejiajia@jmu.edu.cn;) or Lianhuang Li, College of Photonic and Electronic Engineering, Fujian Normal University, Fuzhou 350007, P. R. China; E-mail: [lhli@fjnu.edu.cn;](mailto:lhli@fjnu.edu.cn;) or Jianxin Chen, College of Photonic and Electronic Engineering, Fujian Normal University, Fuzhou 350007, P. R. China; E-mail: [chenjianxin@fjnu.edu.cn](mailto:chenjianxin@fjnu.edu.cn).

**This file includes:**

**Supplementary Table 1:** Characteristics of patients with breast cancers in the training and validation cohorts.

**Supplementary Table 2:** Hazard ratios for 5-year DFS between different histological grades in the three cohorts.

**Supplementary Table 3:** Performance comparison of different models for predicting pathologic grades in validation cohort.

**Supplementary Fig. 1:** MPM images of TILs-1 to TILs-3 in breast cancer. TILs-1 is defined as a pattern of infiltrating lymphocytes surrounded by tumor cells and there are almost no collagen fibers between lymphocytes or collagen fibers are occasionally found in the cancer nest, and the surrounding collagen fibers are sparse and disordered; TILs-2 is defined as a pattern of infiltrating lymphocytes around tumor cells and the fragmented, short collagen fibers exist between lymphocytes; TILs-3 is defined as infiltrating lymphocytes distributed in the TME without direct contact with tumor cells and there are a large number or a small amount of short and discontinuous collagen fibers between lymphocytes, and the surrounding collagen fibers are dense along the same direction. White arrow: tumor cells; yellow arrow: TILs; blue arrow: collagen fibers.

**Supplementary Fig. 2:** Kaplan-Meier curve of 5-year DFS showing the survival difference between G1, G2, and G3 in the training cohort **A**, validation cohort **B** and the whole cohort **C**. G1: Grade1; G2: Grade2; G3: Grade3.

**Supplementary Fig. 3:** **A** Venn software was used to identify the overlapping patients between the actual and predicted in the low risk group of validation cohort. **B** Venn software was used to identify the overlapping patients between the actual and predicted in the high risk group of validation cohort.

**Supplementary Tables:**

**Supplementary Table 1.** Characteristics of patients with breast cancers in the training and validation cohorts.

| **Characteristics** | Training cohort  (n=335) |  | Validation cohort (n=221) | *P-value* |
| --- | --- | --- | --- | --- |
|  |  |  |  |  |
| **Age** |  |  |  | 0.55 |
| ≤50 | 189 (56.4%) |  | 119 (53.8%) |  |
| >50 | 146 (43.6%) |  | 102 (46.2%) |  |
| **Molecular subtype** |  |  |  | 0.774 |
| Luminal A | 81 (24.2%) |  | 53 (24.0%) |  |
| Luminal B | 102 (30.4%) |  | 76 (34.4%) |  |
| HER2-enriched | 78 (23.3%) |  | 46 (20.8%) |  |
| Triple-negative | 74 (22.1%) |  | 46 (20.8%) |  |
| **Tumor size** |  |  |  | 0.107 |
| ≤2cm | 152 (45.4%) |  | 85 (38.5%) |  |
| >2cm | 183 (54.6%) |  | 136 (61.5%) |  |
| **Nodes metastasis** |  |  |  | 0.293 |
| 0 | 180 (53.7%) |  | 110 (49.8%) |  |
| 1-3 | 82 (24.5%) |  | 50 (22.6%) |  |
| ≥4 | 73 (21.8%) |  | 61 (27.6%) |  |
| **Histological**  **grade** |  |  |  | 0.646 |
| Grade 1 | 47 (14.0%) |  | 28 (12.7%) |  |
| Grade 2/3 | 288 (86.0%) |  | 193 (87.3%) |  |
| **ER** |  |  |  | 0.207 |
| Negative | 150 (44.8%) |  | 87 (39.4%) |  |
| Positive | 185 (55.2%) |  | 134 (60.6%) |  |
| **PR** |  |  |  | 0.848 |
| Negative | 167 (49.9%) |  | 112 (50.7%) |  |
| Positive | 168 (50.1%) |  | 109 (49.3%) |  |
| **HER2** |  |  |  | 0.153 |
| Negative | 217 (64.8%) |  | 156 (70.6%) |  |
| Positive | 118 (35.2%) |  | 65 (29.4%) |  |
| **TILs-WG**  **median (IQR)** | 10%  (5%-20%) |  | 10%  (10%-20%) | 0.288 |
| **TILs-score**  **median (IQR)** | 2.122  (1.655-2.575) |  | 2.147  (1.538-2.675) | 0.681 |

Abbreviations: ER, estrogen receptor; PR, progesterone receptor; IQR, interquartile range.

**Supplementary Table 2.** Hazard ratios for 5-year DFS between different histological grades in the three cohorts.

| **Comparison** | Training cohort | |  | Validation cohort | |  | Whole cohort | |
| --- | --- | --- | --- | --- | --- | --- | --- | --- |
|  | HR | 95%CI  *P-value* |  | HR | 95%CI  *P-value* |  | HR | 95%CI  *P-value* |
| **G2 Vs. G1** | 1.935 | 0.9467-3.167  (=0.0759) |  | 0.8386 | 0.3960-1.739  (=0.6222) |  | 1.329 | 0.8167-2.073  (=0.2695) |
|  |  |  |  |  |  |  |  |  |
| **G3 Vs. G1** | 1.97 | 0.9438-3.383  (=0.0755) |  | 1.064 | 0.5220-2.167  (=0.8657) |  | 1.478 | 0.8911-2.299  (=0.1391) |
|  |  |  |  |  |  |  |  |  |
| **G3 Vs. G2** | 1.013 | 0.6633-1.548  (=0.9415) |  | 1.28 | 0.7763-2.142  (=0.3276) |  | 1.116 | 0.8079-1.548  (=0.5018) |
|  |  |  |  |  |  |  |  |  |
| Abbreviations: HR, hazard ratio; G1, Grade1; G2, Grade2; G3, Grade3. | | | | | | | | |

**Supplementary Table 3.** Performance comparison of different models for predicting pathologic grades in validation cohort.

| **Model** | AUC (95%) | SEN (95%) | SPE (95%) | PPV (95%) | NPV (95%) |
| --- | --- | --- | --- | --- | --- |
| **CLI** | 0.694  (0.628-0.754) | 68.39  (61.3-74.9) | 67.86  (47.6-84.1) | 93.6  (89.5-96.2) | 23.7  (18.3-30.2) |
| **TILs-WG** | 0.557  (0.489-0.623) | 32.12  (25.6-39.2) | 85.71  (67.3-96.0) | 93.9  (85.9-97.5) | 15.5  (13.3-18.0) |
| **TILs-score** | 0.752  (0.689-0.807) | 73.58  (66.8-79.6) | 60.71  (40.6-78.5) | 92.8  (89.0-95.4) | 25.0  (18.6-32.8) |
| **Nomogram** | 0.776  (0.715-0.829) | 62.69  (55.5-69.5) | 82.14  (63.1-93.9) | 96.0  (91.6-98.2) | 24.2  (19.9-29.1) |

**Supplementary Figures:**


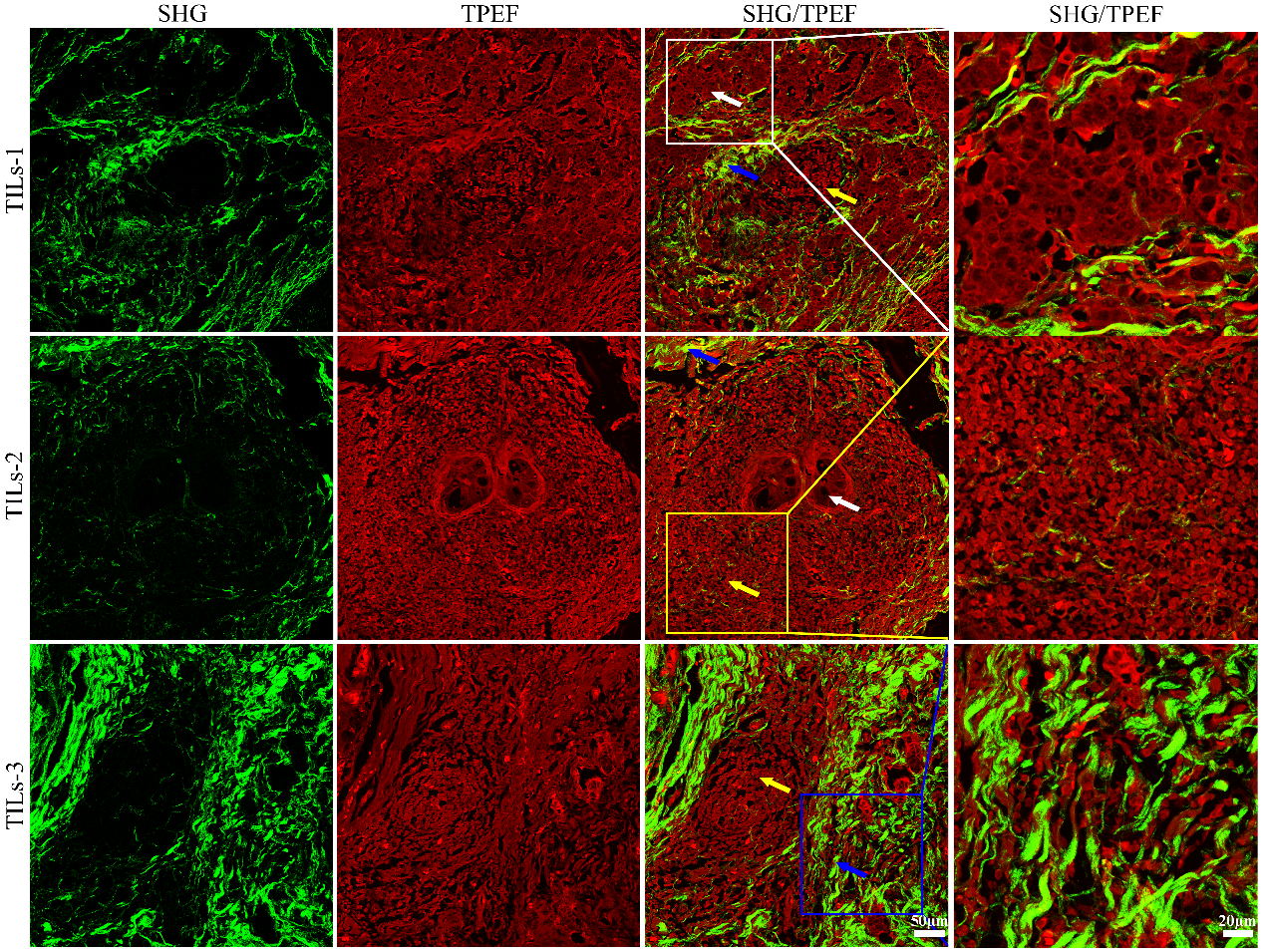


**Supplementary Fig. 1** MPM images of TILs-1 to TILs-3 in breast cancer. TILs-1 is defined as a pattern of infiltrating lymphocytes surrounded by tumor cells and there are almost no collagen fibers between lymphocytes or collagen fibers are occasionally found in the cancer nest, and the surrounding collagen fibers are sparse and disordered; TILs-2 is defined as a pattern of infiltrating lymphocytes around tumor cells and the fragmented, short collagen fibers exist between lymphocytes; TILs-3 is defined as infiltrating lymphocytes distributed in the TME without direct contact with tumor cells and there are a large number or a small amount of short and discontinuous collagen fibers between lymphocytes, and the surrounding collagen fibers are dense along the same direction. White arrow: tumor cells; yellow arrow: TILs; blue arrow: collagen fibers.


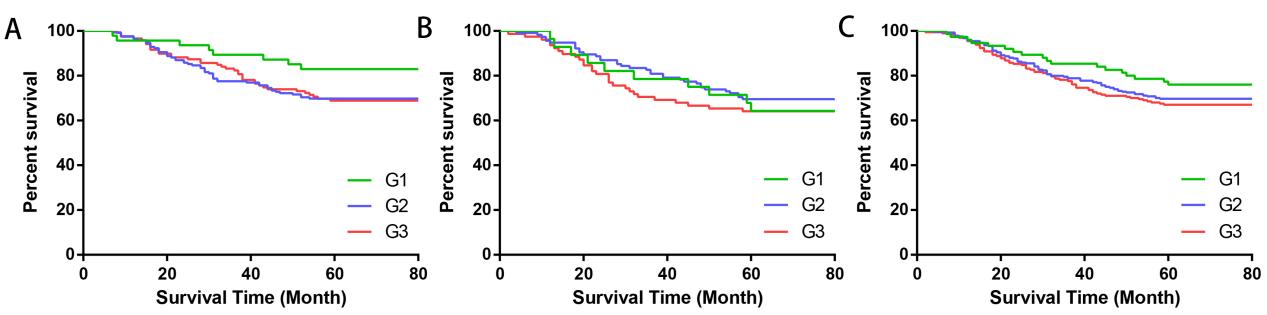


**Supplementary Fig. 2** Kaplan-Meier curve of 5-year DFS showing the survival difference between G1, G2, and G3 in the training cohort **A**, validation cohort **B** and the whole cohort **C**. G1: Grade1; G2: Grade2; G3: Grade3.


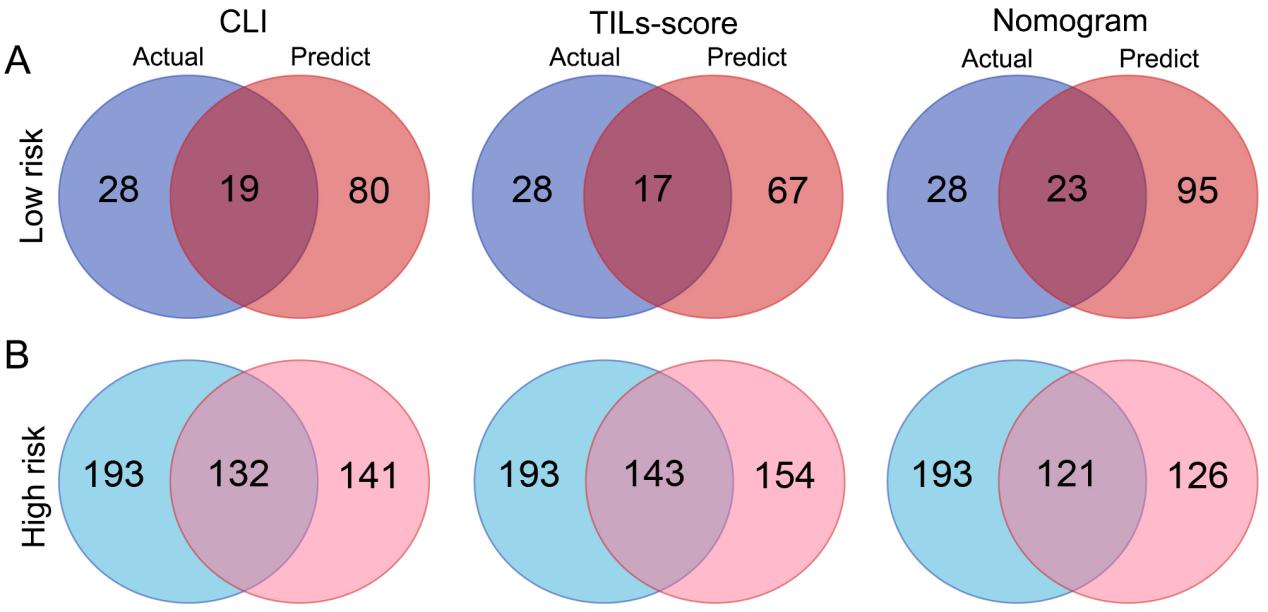


**Supplementary Fig. 3** **A** Venn software was used to identify the overlapping patients between the actual and predicted in the low risk group of validation cohort. **B** Venn software was used to identify the overlapping patients between the actual and predicted in the high risk group of validation cohort.
